# Associations of genetically determined iron status across the phenome: A mendelian randomization study

**DOI:** 10.1371/journal.pmed.1002833

**Published:** 2019-06-20

**Authors:** Dipender Gill, Beben Benyamin, Luke S. P. Moore, Grace Monori, Ang Zhou, Fotios Koskeridis, Evangelos Evangelou, Mike Laffan, Ann P. Walker, Konstantinos K. Tsilidis, Abbas Dehghan, Paul Elliott, Elina Hyppönen, Ioanna Tzoulaki

**Affiliations:** 1 Department of Epidemiology and Biostatistics, School of Public Health, Imperial College London, London, United Kingdom; 2 Australian Centre for Precision Health, University of South Australia, Adelaide, Australia; 3 Institute for Molecular Bioscience, University of Queensland, Brisbane, Australia; 4 South Australian Health and Medical Research Institute, Adelaide, Australia; 5 National Institute for Health Research Health Protection Research Unit in Healthcare Associated Infections and Antimicrobial Resistance, Imperial College London, United Kingdom; 6 Chelsea & Westminster NHS Foundation Trust, London, United Kingdom; 7 Imperial Biomedical Research Centre, Imperial College London and Imperial College NHS Healthcare Trust, London, United Kingdom; 8 Department of Hygiene and Epidemiology, University of Ioannina Medical School, Ioannina, Greece; 9 Centre for Haematology, Imperial College London, United Kingdom; 10 Population Science & Experimental Medicine, Institute of Cardiovascular Science, University College London, London, United Kingdom; 11 Medical Research Council-Public Health England Centre for Environment, School of Public Health, Imperial College London, London, United Kingdom; 12 UK Dementia Research Institute, Imperial College London, London, United Kingdom; 13 Health Data Research UK-London, London, United Kingdom; 14 Population, Policy and Practice, Great Ormond Street Institute of Child Health, University College London, London, United Kingdom; University of Oxford, UNITED KINGDOM

## Abstract

**Background:**

Iron is integral to many physiological processes, and variations in its levels, even within the normal range, can have implications for health. The objective of this study was to explore the broad clinical effects of varying iron status.

**Methods and findings:**

Genome-wide association study (GWAS) summary data obtained from 48,972 European individuals (55% female) across 19 cohorts in the Genetics of Iron Status Consortium were used to identify 3 genetic variants (rs1800562 and rs1799945 in the hemochromatosis gene [*HFE*] and rs855791 in the transmembrane protease serine 6 gene [*TMPRSS6*]) that associate with increased serum iron, ferritin, and transferrin saturation and decreased transferrin levels, thus serving as instruments for systemic iron status. Phenome-wide association study (PheWAS) of these instruments was performed on 424,439 European individuals (54% female) in the UK Biobank who were aged 40–69 years when recruited from 2006 to 2010, with their genetic data linked to Hospital Episode Statistics (HES) from April, 1995 to March, 2016. Two-sample summary data mendelian randomization (MR) analysis was performed to investigate the effect of varying iron status on outcomes across the human phenome. MR–PheWAS analysis for the 3 iron status genetic instruments was performed separately and then pooled by meta-analysis. Correction was made for testing of multiple correlated phenotypes using a 5% false discovery rate (FDR) threshold. Heterogeneity between MR estimates for different instruments was used to indicate possible bias due to effects of the genetic variants through pathways unrelated to iron status. There were 904 distinct phenotypes included in the MR–PheWAS analyses. After correcting for multiple testing, the 3 genetic instruments for systemic iron status demonstrated consistent evidence of a causal effect of higher iron status on decreasing risk of traits related to anemia (iron deficiency anemia: odds ratio [OR] scaled to a standard deviation [SD] increase in genetically determined serum iron levels 0.72, 95% confidence interval [CI] 0.64–0.81, *P* = 4 × 10^−8^) and hypercholesterolemia (hypercholesterolemia: OR 0.88, 95% CI 0.83–0.93, *P* = 2 × 10^−5^) and increasing risk of traits related to infection of the skin and related structures (cellulitis and abscess of the leg: OR 1.25, 95% CI 1.10–1.42, *P* = 6 × 10^−4^). The main limitations of this study relate to possible bias from pleiotropic effects of the considered genetic variants and misclassification of diagnoses in the HES data. Furthermore, this work only investigated participants with European ancestry, and the findings may not be applicable to other ethnic groups.

**Conclusions:**

Our findings offer novel, to our knowledge, insight into previously unreported effects of iron status, highlighting a potential protective effect of higher iron status on hypercholesterolemia and a detrimental role on risk of skin and skin structure infections. Given the modifiable and variable nature of iron status, these findings warrant further investigation.

## Abstract 2

Using Mendelian randomization, Dipender Gill and colleagues investigate the clinical implications of iron levels.

## Author summary

**Why was this study done?:**

- Iron has many vital physiological roles, and variations in its levels can have health implications.
- In this study, we explored the broad clinical effects of varying iron status.

**What did the researchers do and find?:**

- We used randomly allocated genetic variants related to iron status to study its effects on a broad range of medical outcomes.
- Genetic variants related to higher iron status were associated with a lower risk of anemia and hypercholesterolemia and a higher risk of skin and skin structure infections.

**What do these findings mean?:**

- Given that iron status can be modified, the novel, to our knowledge, associations with hypercholesterolemia and skin and skin structure infections could have clinical implications.
- Further research is required to confirm these associations and explore how they can be incorporated towards improving clinical practice.

## Introduction

Iron is a vital nutrient integral to various physiological processes, including metabolism, erythropoiesis, immune function, and cognitive development [1]. Systemic iron status varies considerably, with serum iron having a coefficient of variation of 30.2% in men (mean 21.2 μmol/L, standard deviation [SD] 6.4 μmol/L) and 36.2% in women (18.5 μmol/L, 6.7 μmol/L) [2]. Given the crucial role of iron, deviations in its levels can have notable health implications [1,2]. At the extremes of iron status are iron deficiency and iron overload. Iron deficiency anemia affects 1.2 billion people worldwide and is responsible for 34.7 million years lived with disability per annum [3]. The etiology of iron deficiency includes inadequate dietary iron intake, impaired absorption, increased losses, and increased requirements such as that due to pregnancy [4]. At the other end of the spectrum, iron overload is most commonly attributed to hemochromatosis and iatrogenic iron overload related to blood transfusions, such as in the treatment of refractory anemia or thalassemia [5]. The modifiable nature and wide variation of iron status, both in the healthy population and among individuals with pathologically low or high levels, make the clinical implications of iron status a research priority.

Observational studies into the effects of iron status can be hindered by confounding from unmeasured and unknown environmental factors and reverse causation bias from outcomes that affect iron status. The use of genetic variants related to systemic iron status to study its effects can overcome these limitations because their random allocation during conception minimizes confounding, and their presence from birth prevents reverse causation [6,7]. By studying the effect on iron status related to randomly allocated alleles, such a mendelian randomization (MR) approach has previously been used in targeted analyses to investigate the effect of iron status on risk of Parkinson’s disease, coronary artery disease, and stroke [8–10]. MR can also be applied to traits across the human phenome, in an agnostic exploration termed MR–phenome-wide association study (MR–PheWAS) [11]. Such analysis allows for the rapid and efficient investigation of potential health implications attributable to varying an exposure of interest (such as systemic iron status in this case) and provides direction for further targeted study [12].

In this work, we performed an MR–PheWAS of iron status using data from the UK Biobank. As instruments to study the effect of varying systemic iron status, we used genetic variants concordantly related to serum iron, ferritin, transferrin, and transferrin saturation in a pattern consistent with an effect on overall iron status [8–10,13]. Given the pivotal role of iron across various fundamental physiological processes [1,14] and the opportunity to therapeutically modify systemic levels, the aim of this analysis was to identify a set of health outcomes potentially causally related to iron status. This should guide further clinical research directed towards preventing and treating iron-associated disease.

## Methods

This study is reported as per the Strengthening the Reporting of Observational Studies in Epidemiology (STROBE) guideline (S1 Checklist). Appropriate patient consent and ethical approval were obtained in the original studies from which data for this work were obtained. Although no formal protocol or prospectively documented analysis plan was used in this study, all the main analyses were decided a priori. At the request of the reviewers, only post hoc sensitivity analyses were performed, as described below.

### Genetic instruments for systemic iron status

The exposure phenotype of interest was systemic iron status, which can be measured clinically using the serum iron, ferritin, transferrin, and transferrin saturation biomarkers [15]. We selected genetic instruments for systemic iron status that had relations to these 4 biomarkers in a pattern consistent with an effect on overall iron status, increasing serum iron, ferritin, and transferrin saturation and decreasing transferrin levels [8,10,13,15]. A genome-wide association study (GWAS) performed by the Genetics of Iron Status Consortium on 48,972 European subjects (combined Discovery [N = 23,986] and Replication [N = 24,986] cohorts, 55% female) identified 3 such single-nucleotide polymorphisms (SNPs): rs1800562 and rs1799945 in the hemochromatosis (*HFE*) gene and rs855791 in the transmembrane protease serine 6 (*TMPRSS6*) gene [2,8,10]. Both the HFE and TMPRSS6 proteins have established roles in maintaining iron homeostasis (S1 Text), and therefore variants in their respective genes make viable instruments for systemic iron status [16]. The two SNPs in the *HFE* gene were in low linkage disequilibrium (LD *r*^2^ < 0.01) when considering combined European populations with the LDlink resource [2,17]. All three of these SNPs have previously been shown to be strong instruments for MR analysis as measured by F-statistics > 10 [8,18] and collectively explain approximately 3.8% of the variation in serum iron [2,8]. Genetic association estimates for the 3 iron status instrument SNPs with the 4 biomarkers of iron status (serum iron, ferritin, transferrin, and transferrin saturation), respectively, are provided in S1 Table.

### PheWAS

The PheWAS was performed in the UK Biobank, a prospective cohort study comprising 503,317 individuals aged 40–69 years recruited between 2006 and 2010 [19]. Participants provided blood samples used for genotyping, and their data were linked to Hospital Episode Statistics (HES) from April, 1995 to March, 2016 [19]. PheWAS analysis was restricted to participants of self-reported European descent in order to maintain consistency with the European population used to obtain instruments for systemic iron status. To avoid bias from related individuals, one participant from each pair of relatives was randomly excluded based on a kinship coefficient of >0.0884. We used the International Classification of Diseases (ICD) versions 9 and 10 to identify cases in the HES data, with both incident and prevalent cases included. Self-reported diagnoses were not considered. Diagnoses were aligned to the phecode grouping system in order to optimize identification of clinically relevant phenotypes [20]. Cases were identified as individuals having at least one documented event and controls as individuals with no record of that outcome or its related phecodes [21]. A series of case-control groups were generated for each phecode, and logistic regression analysis was performed for each instrument SNP separately across all phecodes, adjusting for age, sex, genotyping array, and the first 4 genetic principal components. Analysis was limited to phecodes that had 200 or more cases in order to generate improved statistical power for consequent MR analyses (S1 Text) [22,23].

### MR

There is no single biomarker for overall iron status [2], and serum iron levels were used to quantify the genetic associations of the instruments with systemic iron status. PheWAS association estimates for each instrument SNP represent the association of 1 copy of the effect allele with the outcome under consideration. MR estimates for each SNP were calculated as the ratio of this with the corresponding association of the same SNP with serum iron levels (i.e., conventional two-sample ratio method MR) to provide an estimate of the risk of that outcome scaled to a 1 SD increase in serum iron [24]. The SD of serum iron across all individuals included in the Genetics of Iron Status Consortium’s GWAS was 6.1 μmol/L [2]. Standard errors were generated using second-order weights (S1 Text) [24]. Inverse-variance weighted (IVW) meta-analysis of MR estimates for all 3 instrument SNPs was performed to derive the overall MR estimate for the effect of iron status on risk of each considered outcome [18,25]. Statistical significance of MR effect estimates across the considered phenotypes was ascertained using the false discovery rate (FDR) method with a 5% threshold to correct for multiple testing of correlated phenotypes [26].

### Sensitivity analyses

Pleiotropy in the context of MR refers to the phenomenon in which genetic instruments affect the outcome of interest through pathways that are at least partly independent of the exposure under consideration and is a source of potential bias [7,27]. Heterogeneity in the MR estimates generated by different instrument SNPs beyond that expected by chance can be used to indicate the presence of such pleiotropy [28], and we assess for this in our MR–PheWAS analysis using the Cochran Q test (interpreting *P* < 0.05 as evidence of heterogeneity and thus pleiotropy). Only outcomes for which there was no evidence of pleiotropy were taken forward. For such outcomes, sex-stratified IVW MR estimates were also obtained using PheWAS results obtained exclusively from genetically male and female individuals, respectively. Furthermore, MR estimates were also scaled to effects on the ferritin, transferrin, and transferrin saturation biomarkers of iron status.

To further investigate the robustness of the findings to possible pleiotropy, the weighted median MR sensitivity analysis was performed. This orders the MR estimates produced by each instrument SNP by their magnitude weighted for their precision and produces an overall MR estimate based on the median value, with standard error estimated by bootstrapping [29]. It is a robust approach when more than half of the information for the analysis is derived from valid instruments [29].

Statistical analysis was undertaken by DG, BB, GM, and AZ using the software R (version 3.4.2; The R Foundation for Statistical Computing, Vienna, Austria). The TwoSampleMR package was used to facilitate the weighted median MR analysis [30].

## Results

Descriptive characteristics of the UK Biobank participants included in PheWAS analyses, along with the number of phenotypes and cases considered in each disease category, are provided in Tables 1 and 2. Results of the PheWAS and MR–PheWAS for each instrument SNP are provided in S2–S4 Tables, together with the number of cases and controls available for each outcome. After performing exclusions for related and non-European participants, 424,439 individuals were included in the PheWAS analyses, with genetic association estimates for all 3 instrument SNPs available for 904 distinct phecodes. The IVW meta-analysis pooled MR estimates are given in S5 Table, with results of the Cochran Q test for heterogeneity across the 3 SNPs.

**Table 1:** Descriptive characteristics of the UK Biobank participants (N = 424,439) included in PheWAS analyses.

Characteristics	Mean/N (SD/%)
Age, years (SD)	56.8 (8.0)
Sex, female (%)	229,239 (54.0%)
BMI (SD)	27.4 (4.8)
SBP, mmHG (SD)	138.1 (18.6)
DBP, mmHG (SD)	82.2 (10.13)
Current smoker (%)	43,928 (10.4%)

**Abbreviations:** BMI, body mass index; DBP, diastolic blood pressure; PheWAS, phenome-wide association study; SBP, systolic blood pressure; SD, standard deviation.

**Table 2:** The number of phenotypes and cases considered in each disease category.

Disease Category	Phenotypes (N)	Cases (N)			
		Minimum	Median	Mean	Maximum
Circulatory system	98	202	1,048	6,308	133,749
Congenital anomalies	19	211	442	557	1,823
Dermatologic	43	218	799	4,765	82,669
Digestive	116	228	1,455	4,817	79,488
Endocrine/metabolic	49	208	773	4,076	45,303
Genitourinary	106	203	1,376	4,153	103,829
Hematopoietic	22	201	569	2,690	12,759
Infectious diseases	25	219	1,012	2,237	10,752
Injuries and poisonings	59	222	536	1,513	16,683
Mental disorders	36	202	710	3,280	29,405
Musculoskeletal	57	213	925	4,164	53,823
Neoplasms	82	215	1,124	4,261	90,826
Neurological	44	204	567	2,286	40,703
Pregnancy complications	17	208	1,113	1,854	9,534
Respiratory	56	200	1,124	3,837	62,168
Sense organs	64	210	774	2,443	39,998
Symptoms	16	304	2,341	7,036	42,311

For the 19 outcomes reaching statistical significance at the 5% FDR threshold (*P* < 1.1 × 10^−3^), scatter plots representing the SNP–serum iron and SNP–outcome association estimates are shown in S1–S5 Figs. Table 3 details the 14 traits for which there was consistent MR evidence (without suggestion of heterogeneity) across the 3 genetic instruments for a causal effect of higher iron status. Consistent results for these traits were obtained when performing the weighted median MR sensitivity analysis (Table 3) or scaling MR estimates to effects on the different biomarkers of iron status (S6 Table). S7 Table provides the MR odds ratio (OR) per 1 SD increase in genetically determined serum iron level, along with results stratified by sex. Similar estimates were obtained when considering males and females separately, with 95% confidence intervals (CIs) overlapping throughout, although there was some possible suggestion that the association with cellulitis outcomes was stronger for men (S7 Table).

**Table 3:** IVW MR and weighted median MR estimates for the outcomes reaching 5% FDR significance where there was no evidence of heterogeneity as determined by the Cochran Q test.

Phecode	Description	Cases	Controls	IVW MR	IVW MR lower 95% CI	IVW MR upper 95% CI	IVW MR *P*	Weighted median MR	Weighted median MR lower 95% CI	Weighted median MR upper 95% CI	Weighted Median MR *P*
284	Aplastic anemia	12,485	302,401	0.68	0.62	0.74	3.90 × 10^−17^	0.67	0.61	0.74	7.46 × 10^−17^
285	Other anemias	11,586	302,401	0.67	0.61	0.74	9.14 × 10^−16^	0.66	0.60	0.72	3.19 × 10^−20^
280.1	Iron deficiency anemias, unspecified or not due to blood loss	7,340	302,401	0.72	0.64	0.81	3.57 × 10^−8^	0.72	0.64	0.82	7.82 × 10^−7^
281	Other deficiency anemia	8,605	302,401	0.75	0.67	0.83	1.76 × 10^−7^	0.73	0.65	0.82	3.17 × 10^−7^
272.11	Hypercholesterolemia	33,268	285,396	0.88	0.83	0.93	2.07 × 10^−5^	0.89	0.83	0.95	1.01 × 10^−3^
686	Other local infections of skin and subcutaneous tissue	10,784	309,738	1.22	1.11	1.34	5.06 × 10^−5^	1.23	1.13	1.34	9.15 × 10^−6^
689	Disorder of skin and subcutaneous tissue NOS	41,334	280,000	1.10	1.05	1.15	1.11 × 10^−4^	1.11	1.06	1.16	2.78 × 10^−5^
529.1	Glossitis	298	315,742	2.64	1.56	4.46	2.92 × 10^−4^	2.76	1.64	4.66	2.86 × 10^−4^
960	Poisoning by antibiotics	3,446	293,867	0.74	0.62	0.87	4.18 × 10^−4^	0.76	0.65	0.88	5.89 × 10^−4^
681.3	Cellulitis and abscess of arm/hand	5,671	309,738	1.25	1.10	1.42	5.56 × 10^−4^	1.28	1.11	1.47	8.73 × 10^−4^
681.6	Cellulitis and abscess of foot, toe	5,635	309,738	1.25	1.10	1.42	5.56 × 10^−4^	1.28	1.11	1.47	8.73 × 10^−4^
681.5	Cellulitis and abscess of leg, except foot	5,679	309,738	1.25	1.10	1.42	5.79 × 10^−4^	1.28	1.11	1.47	8.73 × 10^−4^
575.6	Cholesterolosis of gallbladder	459	299,761	0.45	0.28	0.72	9.06 × 10^−4^	0.50	0.31	0.81	7.21 × 10^−3^
285.1	Acute posthemorrhagic anemia	262	302,401	0.35	0.19	0.65	9.89 × 10^−4^	0.39	0.20	0.77	1.07 × 10^−2^

The minimum number of cases and controls for any of the 3 genetics instruments are given. **Abbreviations:** CI, confidence interval; FDR, false discovery rate; IVW, inverse-variance weighted; MR, mendelian randomization; NOS, not otherwise specified.

Higher iron status was most negatively associated with risk of acute posthemorrhagic anemia (OR per 1 SD increase in serum iron 0.35, 95% CI 0.19–0.65, *P* = 1 × 10^−3^). In the other direction, higher iron status was most positively associated with glossitis (OR 2.64, 95% CI 1.56–4.46, *P* = 3 × 10^−4^), followed by cellulitis and abscess of the leg, arm/hand, and foot or toe, which all produced similar estimates, OR 1.25 (95% CI 1.10–1.42, *P* = 6 × 10^−4^).

**Fig 1:**
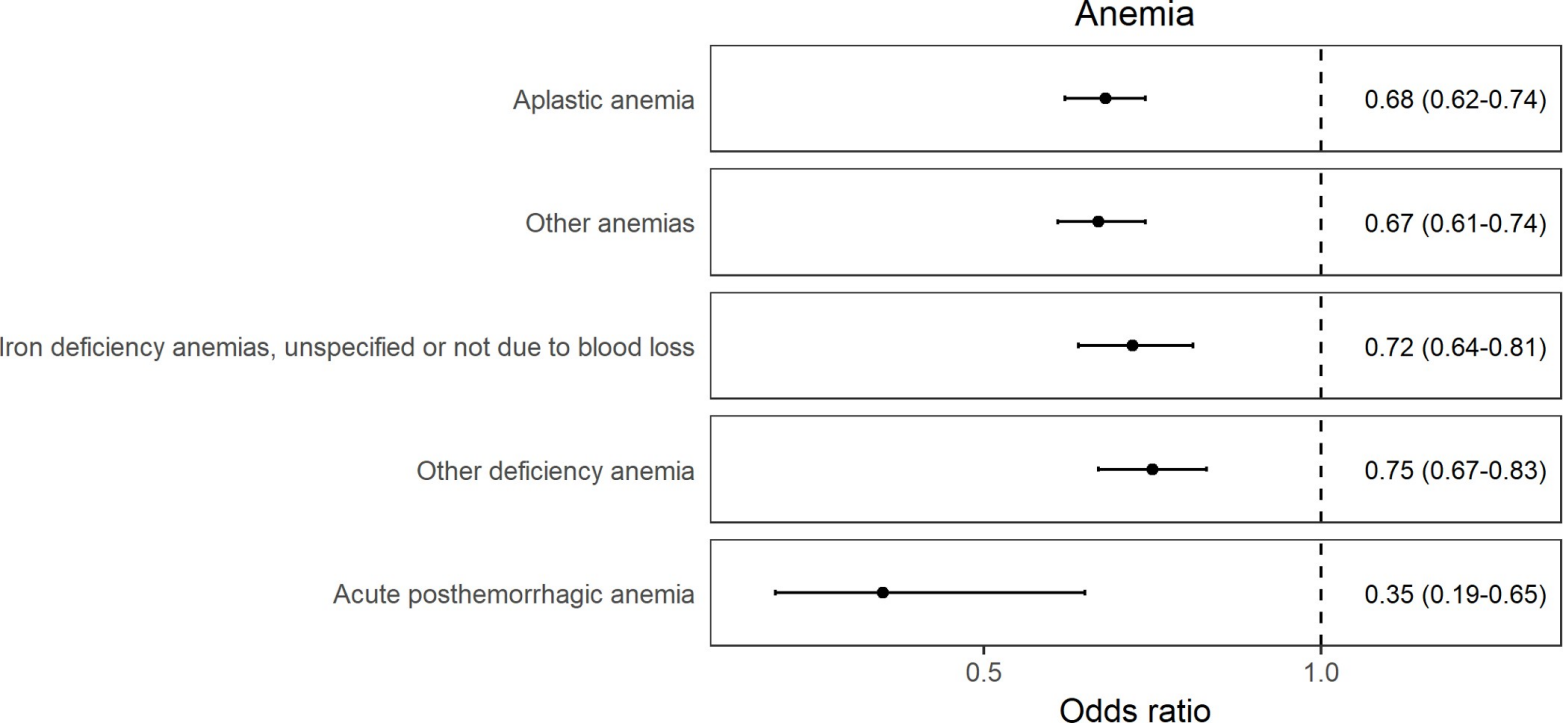
Forest plot of results for traits related to anemia for which there was evidence across the 3 genetic instruments for a causal effect of higher iron status. The ORs are reported as MR estimates corresponding to 1 SD increase in serum iron. MR, mendelian randomization; OR, odds ratio; SD, standard deviation.

**Fig 2:**
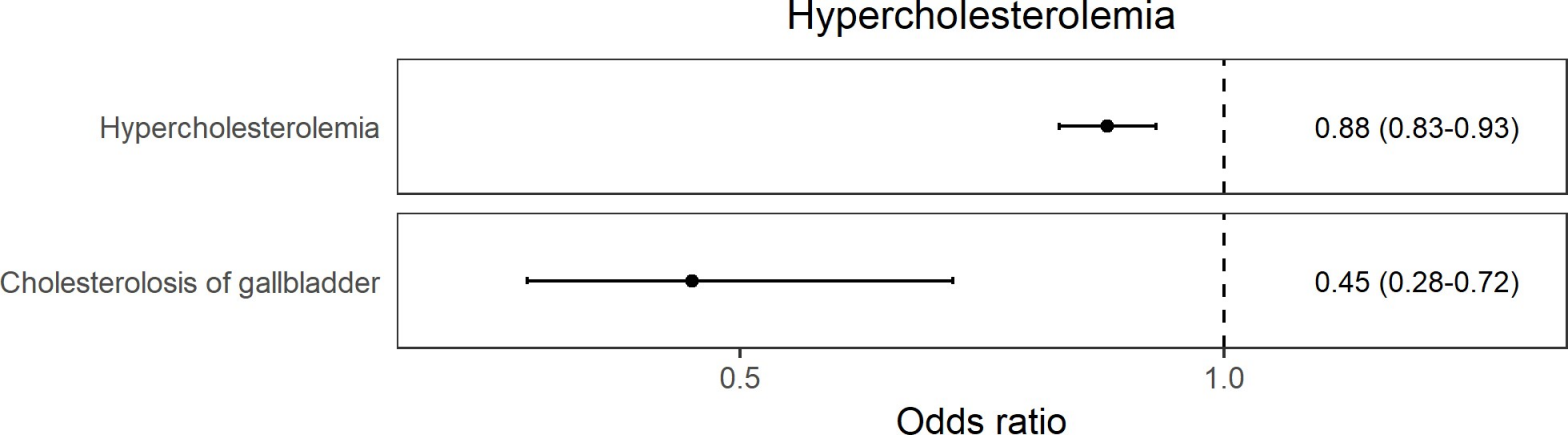
Forest plot of results for traits related to hypercholesterolemia for which there was evidence across the 3 genetic instruments for a causal effect of higher iron status. The ORs are reported as MR estimates corresponding to 1 SD increase in serum iron. MR, mendelian randomization; OR, odds ratio; SD, standard deviation.

**Fig 3:**
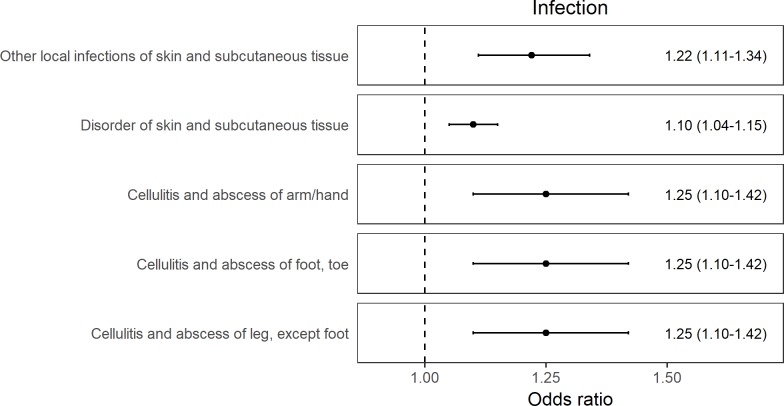
Forest plot of results for traits related to skin and skin structure infections for which there was evidence across the 3 genetic instruments for a causal effect of higher iron status. The ORs are reported as MR estimates corresponding to 1 SD increase in serum iron. MR, mendelian randomization; OR, odds ratio; SD, standard deviation.

The identified effects broadly fall into three categories in relation to higher iron status—outcomes related to decreased risk of anemia (Fig 1), decreased risk of hypercholesterolemia (Fig 2), and increased risk of skin and soft tissue infections (Fig 3). Additionally, there was an association of higher genetically determined iron status with increased risk of glossitis and lower risk of poisoning by antibiotics, which were more difficult to categorize (S7 Table).

## Discussion

In performing what we believe to be the first MR–PheWAS of systemic iron status, we derived evidence of a protective effect of higher iron levels on risk of traits related to anemia, including aplastic anemia, iron deficiency anemia, anemia from other deficiencies, and acute posthemorrhagic anemia. We additionally found evidence of a protective effect of higher iron levels on risk of hypercholesterolemia and cholesterolosis of the gallbladder. In contrast, our MR–PheWAS provided evidence of a detrimental effect of higher iron status on risk of skin and soft tissue infections, including at the hands, arms, toes, feet, and legs.

Consistent with our findings for infection, iron scavenging systems are common among bacteria that infect the skin, such as *Staphylococcus aureus* [31] and *Streptococcus pyogenes* [32]. For these organisms, there is a proposed association between iron metabolism and virulence in human disease [31]. As such, host defense mechanisms act to sequester free iron away from invading pathogens [33]. In the context of abnormalities of iron metabolism such as in hemochromatosis, there is evidence of increased susceptibility to bacterial infections [34], with particular bacterial species showing markedly elevated growth in human serum collected following iron supplementation [35]. Therefore, our finding that higher iron status increases risk of skin and skin structure infections is biologically plausible and consistent with previous evidence. Globally, cellulitis accounted for approximately 598,000 disability adjusted life years across all ages in 2017 [36]. Identifying and modifying potential contributory factors for cellulitis is a global health priority and would potentially serve to decrease antimicrobial use and consequent resistance.

Our findings for higher iron status reducing risk of hypercholesterolemia are also of considerable clinical relevance, with over a third of adult US citizens estimated in 2005–2012 to have cholesterol levels that fall above recommended levels [37], increasing risk of morbidity and mortality related to cardiovascular disease. Similarly, we find evidence that higher iron status decreases risk of gallbladder cholesterolosis, a condition related to the buildup of cholesteryl esters [38]. The *HFE* rs1800562 variant that we use as an instrument for systemic iron status has previously been associated with low-density lipoprotein cholesterol in GWAS meta-analysis [39]. The mechanism underlying this relationship may include effects related to the HFE protein, other variants in close proximity to rs1800562, or systemic iron status. The consistent evidence we identify across two genetic variants of *HFE* (rs1800562 and rs1799945) and one variant of *TMPRSS6* (rs855791) for a causal effect of higher iron status on lowering risk of both hypercholesterolemia and gallbladder cholesterolosis implicates a mechanism in which high iron status more generally affects cholesterol synthesis. In keeping with this, iron status has previously been suggested to affect lipid metabolism in both rats and humans [40,41]. Further work is required to unravel the mechanistic details of any such effect, and the MR technique may be used to investigate the effect of systemic iron status on different lipid fractions, for example.

Previous work has taken an MR approach to investigate the association of genetic variants related to hereditary hemochromatosis with risk of 11 outcomes that are implicated in iron overload [42]. However, the new, to our knowledge, contribution of our current study is that it performed a hypothesis-free investigation into the causal effects of iron status more widely across the human phenome and thus allowed for the identification of novel, to our knowledge, associations, namely potential effects on risk of cellulitis and hypercholesterolemia. Furthermore, our study pooled MR estimates obtained using all available genetic instruments for iron status, rather than focusing on those related to hereditary hemochromatosis [42], and thus better allowed us to investigate the effects of variation in iron status through any cause. The MR methodology used is less vulnerable to environmental confounding and reverse causation than traditional observational research strategies [6,7]. Using genetic variants that are randomly allocated at conception to instrument the effect of modifying systemic iron status [6], we estimated the cumulative lifetime effects of genetically determined variation across over 900 disease outcomes. Our results for higher iron status protecting against iron deficiency anemia and acute posthemorrhagic anemia support the validity of our methodological approach because the role of iron in generating hemoglobin is well-established and associations of our genetic instruments for systemic iron status with red blood cell traits have previously been described [2,8]. A major challenge to such an MR approach is deciphering effects that are attributable to bias related to pleiotropic variants, which we addressed by considering the heterogeneity in the MR estimates produced by our different instrument SNPs [27,43] and in statistical sensitivity analysis using the weighted median MR approach, which is more robust to the inclusion of pleiotropic variants [29]. Furthermore, by using strong instruments associated with serum iron, ferritin, transferrin, and transferrin saturation in a pattern consistent with their effect on systemic iron status, our analysis aims to reflect the effects of varying iron exposure per se rather than that of some other associated traits [8,10].

Limitations of our approach include the use of HES data, which offered a rich source of clinical outcomes that were linked to the genetic data of UK Biobank participants but possibly also introduced misclassification bias [44]. For example, it is unclear whether the observed protective effect of higher iron status on risk of aplastic anemia is attributable to a misclassification of iron deficiency anemia. Similarly, in a scenario in which iron status may not be a cause of aplastic anemia, it may still contribute to diagnosis by shifting borderline cases beyond the requisite threshold for disease label allocation. Our finding of an increased risk of glossitis with higher iron status contradicts the established protective effect of iron on atrophic glossitis, and it is uncertain whether the diagnosis in this context is related to atrophy [45] or superimposed infection, as would be in keeping with our findings for the effect of iron status on risk of superficial infections. For the observed protective effect of higher iron status on risk of hypercholesterolemia (as defined in HES data), the lack of serum cholesterol measures in UK Biobank meant that it was not possible to quantify the magnitude of effect on actual cholesterol levels nor the particular lipid fractions to which this related. Furthermore, ascertainment bias may be responsible for our finding related to poisoning from antibiotics because individuals with higher iron status appear to be more likely to develop particular types of infection (such as those related to the skin and soft tissue), thus potentially also affecting the spectrum of antibiotics to which they are exposed. Finally, inadequate statistical power may have also resulted in false negative results in our MR–PheWAS. The previously described MR effects of iron status on risk of Parkinson’s disease and coronary artery disease (coronary atherosclerosis) were not statistically significant after correcting for multiple testing in our current analysis, although the directions of effect were consistent with previous work (S5 Table) [8,9]. Similarly, type II error and false negative results may have also arisen because of exclusion of all results that evidenced heterogeneity in the MR estimates from different instrument SNPs when attempting to avoid bias from pleiotropy.

Interventions are available to manipulate systemic iron status. The efficacy of oral iron replacement is limited by low gastrointestinal absorption [46], alongside side effects such as abdominal pain, nausea, and constipation that affect around a third of patients and reduce compliance [46,47]. At a population level, fortification of foods with iron-containing micronutrient powders has been efficacious for treating iron deficiency anemia [48, 49]. Anemia refractory to oral supplementation or severe anemia may be managed with intravenous iron infusion [46]. In contrast, iron overload is primarily treated with venesection in hemochromatosis patients [50], with use of iron chelation to enhance iron excretion an additional option [51]. Clinical trials and guidance on the manipulation of iron status have most often related to treatment of anemia [46], such as in the context of menstruation [52], pregnancy [53], and chronic kidney disease [54]. Weaker and more limited evidence exists on the titration of iron status as a treatment for other clinical outcomes, including acute stroke [55], malaria [56], and restless leg syndrome [57]. However, no trial has so far addressed the effect of manipulating iron status to prevent or treat skin and skin structure infections. Similarly, the possibility of targeting iron status to optimize lipid metabolism has not been explored. Given the findings of our study, further research might focus on the degree to which iron status can be titrated in both the prevention and treatment of disease. However, caution must be taken when extrapolating the findings of such MR analyses, particularly because their estimates relate to small variations in iron status within the normal range rather than at extremes of iron deficiency or overload.

In conclusion, this study used MR to explore the effect of iron status across the human phenome and identified a number of novel, to our knowledge, clinically relevant results. Cellulitis and hypercholesterolemia are widespread and of notable significance. Given that iron status is a modifiable trait, further work is warranted to validate our findings, investigate possible underlying mechanisms, and explore whether directed manipulation of iron levels can be used to optimize health outcomes.

## Supporting information

S1 Checklist STROBE checklist. STROBE, Strengthening the Reporting of Observational Studies in Epidemiology.
(DOCX)

S1 Text Roles of HFE and TMPRSS6 in maintaining iron status, statistical power calculation, and second-order weight details. *HFE*, hemochromatosis gene; *TMPRSS6*, transmembrane protease serine 6 gene.
(DOCX)

S1 Table Genetic association estimates for the 3 iron status instruments with the 4 biomarkers of systemic iron status [2]. (XLSX)

S2 Table PheWAS results for rs1800562. All estimates are in log OR units. OR, odds ratio; PheWAS, phenome-wide association study.
(XLSX)

S3 Table PheWAS results for rs1799945. All estimates are in log OR units. OR, odds ratio; PheWAS, phenome-wide association study.
(XLSX)

S4 Table PheWAS results for rs855791. All estimates are in log OR units. OR, odds ratio; PheWAS, phenome-wide association study.
(XLSX)

S5 Table IVW MR estimates for all outcomes. The minimum number of cases and controls for any of the 3 genetics instruments are given. IVW, inverse-variance weighted; MR, mendelian randomization.
(XLSX)

S6 Table Comparison of IVW MR estimates when scaled to 1 SD increase in serum iron, (log10 transformed) ferritin, transferrin saturation, and transferrin. Higher transferrin levels reflect lower iron status. The minimum number of cases and controls for any of the 3 genetics instruments are given. MR estimates are given in log OR units. IVW, inverse-variance weighted; MR, mendelian randomization; OR, odds ratio; SD, standard deviation.
(XLSX)

S7 Table Combined and sex-stratified MR estimates for the effect of iron status. MR estimates are given per SD change in serum iron. MR, mendelian randomization; SD, standard deviation.
(XLSX)

S1 Fig Scatter plot of instrument genetic association estimates for traits related to anemia. (TIFF)

S2 Fig Scatter plot of instrument genetic association estimates for traits related to hypercholesterolemia. (TIFF)

S3 Fig Scatter plot of instrument genetic association estimates for traits related to skin and skin structure infections. (TIFF)

S4 Fig Scatter plot of instrument genetic association estimates for the poisoning by antibiotics and glossitis traits. (TIFF)

S5 Fig Scatter plot of instrument genetic association estimates for the traits in which there was evidence of heterogeneity between instruments. (TIFF)

## Abbreviations

BMI: body mass index
CI: confidence interval
DBP: diastolic blood pressure
FDR: false discovery rate
GWAS: genome-wide association study
HES: Hospital Episode Statistics
HFE: hemochromatosis gene
ICD: International Classification of Diseases
IVW: inverse-variance weighted
LD: linkage disequilibrium
MR: mendelian randomization
NOS: not otherwise specified
OR: odds ratio
PheWAS: phenome-wide association study
SBP: systolic blood pressure
SD: standard deviation
SNP: single-nucleotide polymorphism
STROBE: Strengthening the Reporting of Observational Studies in Epidemiology
TMPRSS6: transmembrane protease serine 6 gene
