# Supplementary material for: Associations of genetically determined iron status across the phenome: A mendelian randomization study
Source: PLoS Med. 2019 Jun 20;16(6):e1002833. doi: 10.1371/journal.pmed.1002833 (PMC6586257; doi:10.1371/journal.pmed.1002833)
Supplement: S1 Text — HFE, hemochromatosis gene; TMPRSS6, transmembrane protease serine 6 gene. (DOCX) [file pmed.1002833.s002.docx]

**Associations of genetically determined iron status across the phenome: A Mendelian randomization study**

# **S1 Text**

## **Roles of HFE and TMPRSS6 in maintaining iron status**

The molecular mechanisms by which the HFE and TMPRSS6 proteins participate in regulation of systemic iron status are complex. While functional pleiotropy cannot be discounted and multiple transcript isoforms of both genes have been identified, their protein expression and functional diversity *in vivo* remain to be defined [1-3].

Under current models, HFE is proposed to play a role in sensing transferrin saturation; it competes with diferric transferrin for binding to transferrin receptor 1, which mediates iron entry into cells by receptor mediated endocytosis [4]. At high transferrin saturations, both HFE and diferric transferrin can bind to transferrin receptor 2, which is required for iron-loaded transferrin induced expression of the iron regulatory peptide hormone, hepcidin [5]. Hepcidin binds to and degrades the only known human exporter of iron, ferroportin, limiting both dietary iron absorption in the duodenum and body iron recycling from macrophages and other cells [6].

TMPRSS6 is a type II transmembrane serine protease which is expressed on the cell surface under low iron conditions [7]. It cleaves hemojuvelin, a co-receptor of bone morphogenetic protein; uncleaved hemojuvelin promotes signaling to increase transcription of *HAMP*, which encodes the precursor of hepcidin [8]. TMPRSS6 may thus inhibit hepcidin production during iron depletion, allowing for increased iron absorption [9].

## **Statistical power calculation**

Statistical power calculations for MR-PheWAS were performed using an online tool available at <http://cnsgenomics.com/shiny/mRnd/> [10]. With an available sample size of 300,000 individuals for genetic association estimates with the outcome, and 3.8% of the variation in the exposure explained collectively by the instruments [11], to achieve a minimum detectable odds ratio of greater than 2 or less than 0.5 with 80% statistical power and a type I error rate of 0.05, approximately 200 cases would be required.

## **Second order weights**

Second order weights were preferred for calculating the standard error of MR estimates, because they account for both error in the exposure and outcome genetic association estimates [12]. The specific formula used was as follows:

$${MR}_{SE}=MR \times\sqrt{\frac{{GX_{SE}}^{2}}{{GX}^{2}}+\frac{{GY_{SE}}^{2}}{{GY}^{2}}}$$

MR is the MR estimate, MR_SE_ is its standard error, GX is the SNP-exposure estimate, GX_SE_ is its standard error, GY is the SNP-outcome estimate and GY_SE_ is its standard error.

## **References**

1. Chelly J, Concordet JP, Kaplan JC, Kahn A. Illegitimate Transcription - Transcription of Any Gene in Any Cell Type. P Natl Acad Sci USA. 1989;86(8):2617-21. doi: DOI 10.1073/pnas.86.8.2617. PubMed PMID: WOS:A1989U231400021.

2. Dion SP, Beliveau F, Desilets A, Ghinet MG, Leduc R. Transcriptome analysis reveals TMPRSS6 isoforms with distinct functionalities. J Cell Mol Med. 2018;22(4):2498-509. doi: 10.1111/jcmm.13562. PubMed PMID: 29441715; PubMed Central PMCID: PMCPMC5867103.

3. Martins R, Silva B, Proenca D, Faustino P. Differential HFE Gene Expression Is Regulated by Alternative Splicing in Human Tissues. PLoS One. 2011;6(3). doi: ARTN e1754210.1371/journal.pone.0017542. PubMed PMID: WOS:000287965200022.

4. Goswami T, Andrews NC. Hereditary hemochromatosis protein, HFE, interaction with transferrin receptor 2 suggests a molecular mechanism for mammalian iron sensing. J Biol Chem. 2006;281(39):28494-8. doi: 10.1074/jbc.C600197200. PubMed PMID: 16893896.

5. Gao J, Chen J, Kramer M, Tsukamoto H, Zhang AS, Enns CA. Interaction of the hereditary hemochromatosis protein HFE with transferrin receptor 2 is required for transferrin-induced hepcidin expression. Cell Metab. 2009;9(3):217-27. doi: 10.1016/j.cmet.2009.01.010. PubMed PMID: 19254567; PubMed Central PMCID: PMCPMC2673483.

6. Nemeth E, Tuttle MS, Powelson J, Vaughn MB, Donovan A, Ward DM, et al. Hepcidin regulates cellular iron efflux by binding to ferroportin and inducing its internalization. Science. 2004;306(5704):2090-3. doi: 10.1126/science.1104742. PubMed PMID: WOS:000225841000062.

7. Zhao N, Nizzi CP, Anderson SA, Wang J, Ueno A, Tsukamoto H, et al. Low intracellular iron increases the stability of matriptase-2. J Biol Chem. 2015;290(7):4432-46. doi: 10.1074/jbc.M114.611913. PubMed PMID: 25550162; PubMed Central PMCID: PMCPMC4326848.

8. Babitt JL, Huang FW, Wrighting DM, Xia Y, Sidis Y, Samad TA, et al. Bone morphogenetic protein signaling by hemojuvelin regulates hepcidin expression. Nat Genet. 2006;38(5):531-9. doi: 10.1038/ng1777. PubMed PMID: 16604073.

9. Silvestri L, Pagani A, Nai A, De Domenico I, Kaplan J, Camaschella C. The serine protease matriptase-2 (TMPRSS6) inhibits hepcidin activation by cleaving membrane hemojuvelin. Cell Metab. 2008;8(6):502-11. doi: 10.1016/j.cmet.2008.09.012. PubMed PMID: 18976966; PubMed Central PMCID: PMCPMC2648389.

10. Brion MJ, Shakhbazov K, Visscher PM. Calculating statistical power in Mendelian randomization studies. Int J Epidemiol. 2013;42(5):1497-501. doi: 10.1093/ije/dyt179. PubMed PMID: 24159078; PubMed Central PMCID: PMC3807619.

11. Gill D, Del Greco M F, Walker AP, Srai SKS, Laffan MA, Minelli C. The effect of iron status on risk of coronary artery disease: a mendelian randomization study. Arterioscler Thromb Vasc Biol. 2017;37(9):1788-92. doi: 10.1161/ATVBAHA.117.309757. PubMed PMID: 28684612.

12. Bowden J, Del Greco MF, Minelli C, Zhao Q, Lawlor DA, Sheehan NA, et al. Improving the accuracy of two-sample summary-data Mendelian randomization: moving beyond the NOME assumption. Int J Epidemiol. 2018. doi: 10.1093/ije/dyy258. PubMed PMID: 30561657.
